# Welcome to *Implementation Science Communications*

**DOI:** 10.1186/s43058-020-00018-4

**Published:** 2020-02-25

**Authors:** Rebecca Armstrong, Anne Sales

**Affiliations:** 1 National Disability Insurance Agency, Melbourne, Australia; 2 Department of Veteran Affairs Centre for Clinical Management Research, VA Ann Arbor Healthcare System, Ann Arbor, Michigan USA; 3 Department of Learning Health Sciences, University of Michigan Medical School, 300N. Ingalls Street, Suite 1161, Ann Arbor, MI 48109-5430 USA

## Abstract

Implementation research in health is a rapidly growing field. Fourteen years after the launch of *Implementation Science*, submissions to the journal have grown exponentially, and the journal now uses a high bar for assessing submitted manuscripts. The field of implementation research in health, however, is growing largely through entry of junior researchers with keen interest in the field whose funding histories and research experience are still developing. We consider it essential to support newer entrants to the field and boundary-spanning work that may consist of smaller, pilot studies, as well as those that contribute primarily descriptive findings.

As a companion journal to *Implementation Science*, *Implementation Science Communications* will accept a broad and diverse range of article types, and provide an important platform for smaller scale or more descriptive research. As with *Implementation Science*, no specific discipline, research design, or paradigm will be favoured.

## Background

*Implementation Science* launched 14 years ago, as a new journal for a relatively new, emerging field. Its growth has been rapid and in many ways, quite spectacular. As it has evolved, and as the field of implementation science in health has matured, a number of important issues have arisen.

First, the decision was made to narrow the scope of the journal to the field of implementation research in health. While this decision has been ratified by the continued increase in the number of submissions annually, and the growth in the number of papers published, it has resulted in the exclusion of related domains such as social services or education.

Second, over the last several years, requirements for reports that describe rigorously designed studies have increased, as a result of editorial decisions, documented about every 2 years by editorials detailing common reasons for rejection from *Implementation Science* [1–3]. The net cumulative effect of these decisions, while welcomed by many in the field, has been to increase the focus on large, well-funded studies being published in *Implementation Science.*

Finally, as the field has continued to grow and emerge, new frontiers are being opened up. Many of these are in low- or middle-income countries, where there has been an explosion of implementation science studies over the last decade. Others are in related or adjacent fields, including studies focusing on health outcomes in educational or social service systems. The field of public health, which has different meanings and contexts in different countries, has embraced implementation research, and built on it with a stronger emphasis on community oriented approaches. Digital health has become well established, and methods of melding implementation research with digital health and the steadily increasing array of digital data sources are being rapidly explored. New developments often begin with smaller scale, pilot studies.

All of these important developments require outlets for publication. Many established journals are moving rapidly to engage with the implementation science research community, while new journals are being established as well. In this rapidly expanding landscape, we felt that it was helpful to launch another outlet, closely linked to our flagship journal. The close linkage supports the growth of knowledge, and tracking new knowledge, in this rapidly evolving field.

Welcome to *Implementation Science Communication*s! This journal has been launched to capture the breadth of work being undertaken in this burgeoning field. The recently published editorial with our *Implementation Science* colleagues outlined many of the reasons why we developed this new journal [2]. As a team, we have a commitment to supporting both rigorous and exploratory approaches to understanding and evaluating implementation of healthcare interventions.

## Restating our scope

We recommend that individuals interested in submitting to this journal review the most recently published editorial [2]. This sets out differences between the journals and will help authors to navigate where their paper best sits. We used this previously published editorial to highlight key differences between *Implementation Science* and *Implementation Science Communications*.

A few key points are important to emphasise. *Implementation Science Communications* maintains a commitment to sound science. We will accept a range of study designs including case studies, smaller pilot studies, pre-implementation studies, or descriptive studies [2]. While we do not require the level of rigor necessary for publication in *Implementation Science* (e.g. randomised controlled trials, multiple sites), manuscripts must use appropriate and sound methodological approaches. They must also incorporate a clear focus on implementation methods and outcomes.

We are particularly interested in manuscripts which:

- Apply theory and its relevance in general or niche settings
- Explore de-implementation
- Examine implementation in policy settings
- Describe new developments and build knowledge about the use of digital health interventions
- Document implementation interventions within single hospital/health service settings, or smaller samples of settings, especially pilot studies to help design larger scale studies
- Explore implementation in community-based settings, social services, and education
- Reports of studies from low- and middle-income country settings

## Journal content and features

Similar to *Implementation Science*, we are interested in publishing a range of papers. These include original research, debates, systematic reviews, methodologies, and short reports. *Implementation Science* has a strong reputation for expedited editorial processes wherever possible. We will maintain this approach, particularly through a rapid initial triage process.

Since launching the journal web site and opening for submissions in July, we have received over 80 submissions. These have been a blend of research reports, protocol papers, and other article types. This first issue of *Implementation Science Communications* reflects some of the diversity of the submissions we have received, with more to come in the future. In this inaugural volume, protocol papers are heavily featured, in part because they are already peer-reviewed when submitted. We expect to include at least two or three research reports among our launch articles, but in future months, we will also include short reports, systematic reviews, and other types of papers.

## Introducing our editorial team

We welcome a highly skilled team of Associate Editors to support the work of the journal. Their profiles are available (https://implementationsciencecomms.biomedcentral.com/about/editorial-board/editorial-board-profiles). During 2019, we sought expressions of interest to join us as either Associate Editors or a member of our Editorial Board. In short-listing for Associate Editors, we sought a mix of expertise in discipline, geography, and experience in implementation science (including theory, methods, intervention design, evaluation). We are thrilled with our recruitment and the work that has been undertaken by the team in the lead-up to this first edition of *Implementation Science Communications*. The team have a mix of policy, health services, public health, and clinical perspectives.

## Peer review

Our commitment to open science (with open peer review) continues in *Implementation Science Communications*. As with all journals, we continue to seek the support of the academic, policy, and practice communities to support us in our peer review processes. We have deliberately decided to share the reviewer database between *Implementation Science* and *Implementation Science Communications* for three reasons. First, there is a limited community of expert reviewers in this area. Second, both journals have a commitment to experimentation with forms of review, and methods of mentoring researchers new to this field in review processes and standards. Third, we have routes for direct transfer between *Implementation Science* and *Implementation Science Communications*, which are bidirectional. Most transfers come from *Implementation Science* to *Implementation Science Communications*, but we have also transferred one manuscript the other way to date.

Stay tuned for more information about plans for this experimentation!

## Conclusions

We are excited about launching this new journal and providing opportunities for publication of original research, methodological discussions, and debates that ultimately expand our knowledge of implementation science. We hope this journal fills an important gap in the publication of implementation science research.

## Data Availability

Not applicable
